# Performance-based Incentives to Improve Health Status of Mothers and Newborns: What Does the Evidence Show?

**Published:** 2013-12

**Authors:** Rena Eichler, Koki Agarwal, Ian Askew, Emma Iriarte, Lindsay Morgan, Julia Watson

**Affiliations:** ^1^Broad Branch Associates; ^2^JHPIEGO; ^3^Population Council; ^4^Inter-American Development Bank; ^5^DFID

**Keywords:** Health systems strengthening, Pay for performance (P4P), Performance-based financing (PBF), Provider payment mechanisms, Results-based financing (RBF)

## Abstract

Performance-based incentives (PBIs) aim to counteract weak providers’ performance in health systems of many developing countries by providing rewards that are directly linked to better health outcomes for mothers and their newborns. Translating funding into better health requires many actions by a large number of people. The actions span from community to the national level. While different forms of PBIs are being implemented in a number of countries to improve health outcomes, there has not been a systematic review of the evidence of their impact on the health of mothers and newborns. This paper analyzes and synthesizes the available evidence from published studies on the impact of supply-side PBIs on the quantity and quality of health services for mothers and newborns. This paper reviews evidence from published and grey literature that spans PBI for public-sector facilities, PBI in social insurance reforms, and PBI in NGO contracting. Some initiatives focus on safe deliveries, and others reward a broader package of results that include deliveries. The Evidence Review Team that focused on supply-side incentives for the US Government Evidence Summit on Enhancing Provision and Use of Maternal Health Services through Financial Incentives, reviewed published research reports and papers and added studies from additional grey literature that were deemed relevant. After collecting and reviewing 17 documents, nine studies were included in this review, three of which used before-after designs; four included comparison or control groups; one applied econometric methods to a five-year time series; and one reported results from a large-scale impact evaluation with randomly-assigned intervention and control facilities. The available evidence suggests that incentives that reward providers for institutional deliveries result in an increase in the number of institutional deliveries. There is some evidence that the content of antenatal care can improve with PBI. We found no direct evidence on the impact of PBI on neonatal health services or on mortality of mothers and newborns, although intention of the study was not to document impact on mortality. A number of studies describe approaches to rewarding quality as well as increases in the quantities of services provided, although how quality is defined and monitored is not always clear. Because incentives exist in all health systems, considering how to align the incentives of the many health workers and their supervisors so that they focus efforts on achieving health goals for mothers and newborns is critical if the health system is to perform more effectively and efficiently. A wide range of PBI models is being developed and tested, and there is still much to learn about what works best. Future studies should include a larger focus on rewarding quality and measuring its impact. Finally, more qualitative research to better understand PBI implementation and how various incentive models function in different settings is needed to help practitioners refine and improve their programmes.

## INTRODUCTION

Health financing strategies that incorporate performance-based incentives (PBIs) are being applied in many developing countries, often with improvements in the health of mothers and newborns as the central goal. Defined as “any program that rewards the delivery of one or more outputs or outcomes by one or more incentives, financial or otherwise, upon verification that the agreed-upon result has actually been delivered” (1), PBI is being implemented to strengthen the linkages between funding for health and health outcomes and to stimulate actions by households, providers, and other health system actors to overcome obstacles in order to achieve health outcomes. This paper focuses on the impact of PBI initiatives that pay providers (individuals and facilities) and their supervisors (at subnational levels of government) if they achieve predetermined performance measures of the health of mothers and newborns. It is based, in part, on lessons that emerged from the US Government Evidence Summit on Enhancing Provision and Use of Maternal Health Services through Financial Incentives, which took place in Washington, DC in April 2012. While evidence of the impact of PBI is not fully clear, this paper will argue that lack of evidence may be due to a dearth of quality studies rather than weak programmes or inadequate impact of PBI schemes. We present the available evidence and suggest options for enhancing the global evidence base.

The imperative to reduce maternal mortality in the developing world has increased political and financial commitments and spurred momentum around Millennium Development Goal 5 (MDG 5), which calls for reduction by three-fourths the maternal mortality ratio and achieving universal access to reproductive health services between 1990 and 2015. In total, 287,000 women die each year from complications of childbirth, the vast majority of whom live in developing countries. In sub-Saharan Africa, women have a lifetime risk of maternal death of 1 in every 39 compared to 1 in every 3,800 in developed regions of the world—the largest difference between poor and the rich countries of any health indicator (2). Much good has been done but experience has shown that health systems continue to underprovide proven cost-effective interventions and fail to reach the poorest populations. PBI is a promising intervention that, by changing incentives for many people who together comprise a health system, may contribute to reducing maternal mortality.

Ensuring maternal wellbeing and survival requires knowledge of and demand for services by women and multiple interactions with a functioning health system capable of delivering quality reproductive and specifically maternal health services. Individuals must know when to seek care and demand services; health workers must be motivated to deliver care of sufficient quality; and the institutions they work for must be encouraged and enabled to make the systemic changes required to achieve the health goals for mothers and newborns. While maternal mortality is partly caused by insufficient inputs, such as equipment, supplies, facilities, trained health workers, and a coordinated referral system, it is also driven by disincentives in the health system that can hinder delivery of interventions that improve the health of women and their babies. The choices providers and their supervisors make depend on what they have (that is, on inputs), what they know, and, critically, on what influences and motivates them. PBI initiatives aim to counteract dysfunctional incentives and drive changes that strengthen health systems and improve outcomes.

Translating funding into health outputs or outcomes requires many actions by a large number of people that span from the national to the community level, from officials in the national ministry of health to people who work on the supply chain for lifesaving commodities and to health workers and their supervisors at the district level and in communities. Each of these people encounters internal drivers and extrinsic incentives that are both financial and non-financial, and these incentives interact to induce behaviours that contribute to either good or bad health outcomes. In many challenging contexts, existing incentive environments do not sufficiently inspire and reward the amount and/or type of actions needed to achieve the desired results. At the service-delivery level, for example, salary structures that provide relatively low, fixed salaries that do not vary with performance may not spur providers to take the steps necessary to attract patients or proactively solve bottlenecks. This may also lead to low productivity, absenteeism, poor quality, or lack of innovation. District health supervisors may provide minimal support partly due to their low salaries and weak accountability structures, which may not motivate them to do all they can to assure that the population in their district has access to quality services.

PBI aims to counteract weak incentives in health systems of many developing countries by providing rewards directly linked to actions that contribute to health outcomes. Supply-side incentives can be given to healthcare providers when they achieve performance targets (such as providing the final course of malaria prophylaxis to a specified percentage of pregnant women in a given region) or a unit fee for providing each unit of a prioritized service. In addition, performance-based incentives are sometimes provided to health managers at the district, provincial, and national levels, conditional on such things as uninterrupted supplies of lifesaving commodities and the quality of services delivered at the facilities they are responsible for.

PBI is not only a financing strategy. It can also have a significant impact on health systems. For example, because PBI schemes pay for results, the success of schemes rests fundamentally on the ability to monitor and verify those results accurately. Monitoring and verification require the development of robust health information and management systems; so, incorporating the PBI concept, even into the programmes aimed at specific diseases, can reinforce efforts to improve the timeliness, credibility, and accuracy of national reporting and monitoring. PBI can enhance the effectiveness of human resources for health by stimulating innovation, enhancing motivation and team work, incentivizing service in remote locations, and improving retention. Arrangements in governance can be strengthened by holding facilities, district teams, and health committees accountable for results. By rewarding increases in the quality of services as well as incentivizing increases in quantities of priority services, PBI can strengthen service delivery. Some schemes provide examples of innovative solutions to the persistent challenge of assuring reliable supplies of lifesaving medicines at the service-delivery level.

Supply-side PBI programmes have been introduced allover the world, supported by governments and their donor partners. The World Bank, Norway, DFID, and USAID have been particular leaders in this field. A review of PBI programmes focused on maternal health (2) shows that there are 24 documented programmes worldwide, ranging from performance-based aid programmes, supply-side programmes, performance-based contracting; and on the demand side, voucher schemes and conditional cash transfers (CCTs). Many begin as donor-funded pilots while some are scaled up nationally and funded with a combination of domestic and donor resources (e.g. Argentina and Rwanda).

Despite the wealth of PBI experience that has accumulated over the last decade, evidence of impact is just beginning to become available. As described in more detail below, the available evidence suggests that incentives that reward providers for institutional deliveries result in an increase in the number of institutional deliveries. There is some evidence that the content of antenatal care can improve with PBI but there is limited evidence of impact on increasing the number of women who receive at least four antenatal care visits. We found no direct evidence of the impact of PBI on neonatal health services or on the mortality of mothers and newborns. A number of studies describe approaches to rewarding quality as well as increases in the quantities of services provided.

The details of PBI schemes are complex, making it difficult for researchers to include enough detail in journal articles to inform adaptation of a successful scheme in another setting. The focus of this paper is on whether there is evidence that PBI contributes to the use of maternal and neonatal health services and their quality. None of the reviewed papers includes enough information on the specifics of critical programme details, such as indicators, how and whether targets are set, how quality is assessed, the terms of payment, how results are monitored and verified, and how schemes are administered.

## MATERIALS AND METHODS

This paper reviews evidence from published and grey literature that spans PBI for public-sector facilities, PBI in a social insurance reform, PBI in NGO contracting, and PBI in targeted safe delivery schemes. In addition, relevant evidence from multicountry reviews (2,3) is cited. The Evidence Review Team (ERT) for the US Government Evidence Summit on Enhancing Provision and Use of Maternal Health Services through Financial Incentives, reviewed 32 research reports and papers categorized in terms of incentive mechanism: performance-based incentives (PBI), user-fees, or insurance, that were provided by the team that conducted the literature reviews (4). A discussion of the literature review approach is provided in another paper in this volume. In addition, we added a paper from the grey literature on a safe-delivery scheme in Bangladesh (5) and cross-country studies that contributed to understanding of context, design, and implementation issues (2,3). Table 1 provides an overview of countries covered, scale, timeline, and evaluation method.

## RESULTS

Nine country-specific papers under review that incorporated PBI include the following:

- Two incorporate PBI for public-sector facilities [Democratic Republic of Congo (DRC) (6) and Rwanda (7)]
- One incorporates PBI as part of a social insurance reform [Egypt (8)]
- Three incorporate PBI into the way NGOs are paid [Afghanistan (9), Cambodia (10), and Haiti (11)]
- Three incorporate PBI into safe-delivery schemes that include both supply- and demand-side incentives [Bangladesh (5), Nepal (12), and the Philippines (13)]

The majority of reviewed papers had methodological weaknesses and gaps that generate more questions than answers. Moreover, in the majority of cases, insufficient details are presented on the actual incentive model to be able to consider its replicability. Three studies included before-after designs (10,12,13), using baseline and endline data; the design of these assessments is typically too weak to disentangle the effect of the incentive from other contextual factors. In some cases, comparison or control groups are also used (5,6,8,9); and one paper applied econometric methods to a five-year time series (11). Only one paper reported results from a large-scale impact evaluation with randomly-assigned intervention and control facilities (7).

**Table 1.** Features of PBI studies

Country	Scale	Duration	PBI type	Study design
Afghanistan (9)	3.6 million population coverage in intervention; 10.7 million population coverage in comparison locations	4 years	Performance-based contracting of NGOs to manage health services at the province level	Natural experiment that compares NGO performance in regions with PBI to regions with contracts that do not include PBI
Bangladesh (5)	4 districts: 1 as control; 2 as study arm 1; 1 as study arm 2	14 months	Supply-side facility-based PBI compared to combination of demand-side and supply-side PBI	Separate sample pre-post design with three arms: control, supply plus demand-side incentives, supply-side incentives alone
Cambodia (10)	12 districts	3.5 years	After service delivery was contracted out to NGOs, this study looked at change in performance of moving back to public service delivery with contracted-in management	Comparison of baseline and endline data
Democratic Republic of Congo (6)	4 districts: 2 treatment, 2 control	2 years	Performance-based financing at the facility level	Intervention and control comparisons
Egypt (8)	5 governorates	1 year	Performance-based incentives to facilities paid by a social insurance scheme	Intervention and control comparisons
Haiti (11)	2.7 million population coverage	6 years	Performance-based contracts with service delivery NGOs	Phased graduation of NGOs into PBI allowed econometric analysis to assess impact
Nepal (12)	1 district	7 years (5 years before and 2 years after)	Performance-based payments to facilities for each delivery attended at facility or woman's home	Before-after evaluation using baseline and endline data
Philippines (13)	5 provinces	2 years	Performance-based incentives to both women and community health workers who accompany women to deliver in health facilities	Before-after evaluation using baseline and endline data
Rwanda (7)	166 facilities: 80 intervention, 86 controls	25 months	Performance-based financing to intervention facilities. Fees for a list of specified services plus a quality tool that result in a score that deflates fees	Large-scale impact evaluation with randomly-assigned intervention districts that were implemented in phases, allowing late entrants to act as controls for the initial-phase entrants

In a 25-month study in Rwanda that employed the most rigorous evaluation methods among studies included in this review, 166 facilities were randomly assigned to receive payments for performance (n=80) or to act as controls (n=86). To isolate the effect of the performance-based incentive to intervention facilities, control facilities received an additional unconditional budget transfer equivalent to the average amount of performance-based payments earned by intervention facilities (7). Intervention facilities could earn fees for each unit of provided maternal, child and infectious disease services included on the output-based fee-list. To incentivize improvements in quality, facilities were assessed each quarter, using a quality tool that resulted in a score that served to deflate the fees earned from quantities of services provided. Poor quality and an associated low score could result in a considerable reduction in the fees a facility could earn while good quality could ensure that a facility earned close to all fees for the quantity. In comparison, control facilities received a quarterly budget transfer equivalent to the average amount the intervention facilities earned as performance bonuses. This model allowed the researchers to address the question of whether improved performance was due to additional funds or to the performance-based financing system that conditioned payment on results. Compared to controls, women living in the catchment areas served by intervention facilities had a significant 23% higher probability of delivering in a health facility than women living in the regions served by control facilities. There was no significant difference between the intervention and the control facilities in terms of the number of women receiving at least four antenatal care (ANC) visits but intervention facilities showed a significant increase over controls in the quality of ANC as measured by a composite quality index and provision of tetanus toxoid vaccine (7).

Inspired by the generally positive results from Rwanda, a number of countries in Africa are either considering or have begun implementing PBI as a complement to input-based financing. Learning from Rwanda has motivated countries to layer performance-based incentives on top of the historical way public and many NGO services are financed that pay for the inputs needed to produce health services, such as salaries and medicines but not for the results produced. We see a combination of fees to facilities for each delivered unit of specified services, complemented by a quality assessment that results in a score that either deflates (Rwanda) or inflates (Burundi, DRC) payments in facility-based performance (2).

In the Democratic Republic of the Congo (DRC), which has implemented a number of PBI mechanisms over the last decade, a small and less rigorous study in South Kivu did not find any association between PBI and the number of institutional deliveries or ANC, although authors report that a household survey showed improved patient-perceived quality of care and availability of medicines in PBI facilities compared to households that accessed control facilities (6). The PBI model in the DRC is similar to that in Rwanda as it rewards fees for a list of services combined with a quality assessment that results in a score. However, in the DRC, this score inflates fees in contrast with the model in Rwanda that uses the quality score to deflate fees. The study also reported that intervention facilities were able to address shortages of medicines, which resulted in fewer stock-outs than experienced by the control facilities. The performance-based payments provided liquid funds to procure essential medicines from local suppliers.

Egypt and Argentina offer worthwhile lessons on how to incorporate performance-based incentives into the way social insurance schemes pay providers. In the context of reforms that created a social insurance fund aiming at enhancing access to and use of a basic benefit package, including maternal/newborn care and family planning, Egypt offered performance-based incentives for achieving performance targets as part of a new delivery model for primary care that centred on family doctors. A one-year study comparing intervention facilities that received performance-based incentives with control facilities that received fixed (unconditional) salary top-ups found that the quality of family planning and ANC was better in PBI facilities as measured by more complete medical histories, more follow-up visits, and more follow-up tests (8). Results are expected in 2013 from an impact evaluation of the expansion of an insurance scheme for poor women and children in Argentina. The scheme transfers funding from federal level to provinces, based partly on the number of eligible individuals enrolled in a maternal and child health insurance programme called Plan Nacer, and partly based on performance on 10 indicators, five of which address maternal health, and the sixth which focuses on counselling for sexual and reproductive health (2).

In contexts with disrupted health sectors where NGOs are prominent providers, evidence on incorporating conditions into contracts with service-delivery NGOs that link a portion of funding to health results suggests a positive impact on the number of institutional deliveries and less clear impact on other rewarded indicators, such as ANC visits and postnatal care visits. For example, beginning in 1999, a USAID-funded programme in Haiti that contracts NGOs to deliver health services, phased in PBI that rewarded attainment of population coverage targets with a subset of supported NGOs each year. This multi-year dataset offers the opportunity to compare the performance of the same NGO before and after entry into the performance-based contracting as well as to examine the performance of NGOs under both payment regimes in a given year. Results suggest that being paid based on performance was associated with a highly significant 17-27 percentage point increase in attended deliveries (11). Adding contract period effects to regressions with NGO-specific fixed effects erode the impact on three or more ANC visits, suggesting that some of the improved performance was due to NGO's characteristics rather than the incentive.

Performance-based contracting in Afghanistan offered NGOs that received contracts to oversee and deliver care in an entire province the opportunity to earn performance bonuses based on the score achieved on a “balanced score card” and on achieving service-delivery targets. The balanced score card tracks performance on multiple indicators within six domains: patients’ satisfaction, human resources for health, facility's capacity for service provision, financial management, socioeconomic equality, and gender equity. While the PBI model outperformed other schemes in Afghanistan that funded NGOs with expenditure-based reimbursement on measures, such as attended deliveries and antenatal care, it is difficult to attribute this performance to PBI because there were other important differences in the context and security situation that were not controlled for (9).

As part of contracting out management of public service delivery in Cambodia, financial awards were given to facilities and teams based on attainment of performance targets for ANC, facility deliveries, and contraceptive acceptance. Incentives were distributed to facility staff in a three and-a half-year study that showed an increase in institutional deliveries but no change in uptake of contraceptives (10).

PBI programmes that specifically target safe deliveries appear to be expanding in South Asia (Bangladesh, India, and Nepal) and beyond (Philippines). A range of individuals may receive performance-based payments in these programmes: the eligible women who deliver in a health facility; community health workers who accompany the women; and, in some settings where the provider performs the delivery. Note that another paper in this JHPN Supplement presents evidence of the impact of similar programmes that use physical vouchers to enable women (often identified as poor) to receive care from accredited providers who are also paid fees to serve voucher-holders (14).

Nepal introduced a safe-delivery programme that complemented a conditional cash transfer to women, with providers’ incentives that rewarded either attending deliveries at homes or in facilities (12). A study of the programme traced the uptake of professional delivery care in one district over a seven-year period (five years before introduction of the intervention and two years after), with no control or comparison group and found an increased likelihood of delivery in a government facility. However, in communities with organized women's groups, this increase was significant while, in communities without women's groups, no impact was seen, causing researchers to question whether the influence of women's groups was the driver of change instead of the PBI (12).

A study in Bangladesh compared the impact on safe deliveries over a 14-month period under three regimes: 1 control district, 2 districts with a combined incentive model that provided performance-based payments to women and to facilities, and 1 district where performance-based payments were provided exclusively to facilities (5). In each district, baselines were established for quantities of antenatal care, family planning, institutional deliveries, and postnatal care services. Using these baselines, two levels of target increase were set that calculated successive 20% increase over the baseline levels. Baselines were also established for quality, using a 100-point scoring system that covered MNCH service units, including the emergency room, antenatal care and family planning corner, obstetrics room, labour room, operation theatre, paediatrics ward, store, pharmacy, laboratory, scrub room, and autoclave room. To receive performance-based payments, facilities needed to achieve targets for both quality and quantity. The combined incentive model had a larger effect on quantities of women receiving antenatal care, normal deliveries, postnatal care, and on the quality of care than providing incentives to women alone. On average, deliveries in health centres doubled and, in district hospitals, the rate grew 38% (5).

A project in the Philippines provides incentives to Women's Health Teams to track and counsel all women in their catchment area and provides a payment for each pregnant woman they accompany for facility-based deliveries. A small study, with no controls, reported a 7.6% increase in facility-based deliveries over the previous period but it was unclear how much of the increase could be attributed to the supply-side incentive or the demand-side incentive as the women also receive a payment (13).

Table 2 presents the indicators that are incentivized to increase the numbers of deliveries and antenatal care and to improve quality in each study. Impact on these indicators is presented when available and relevant.

## DISCUSSION

The majority of studies had weaknesses, although it is not clear from the papers themselves if the actual programmes were poorly designed or implemented, or the evaluation studies were weak. For example, the way in which quality is defined and monitored is not always clear; so, it is difficult to conclude whether PBI schemes improve the quality of performance measures of clinical care known to influence health outcomes. While most studies demonstrated an increase in the number of deliveries with a skilled attendant, we know from recent studies on observed quality of care that even skilled providers do not perform according to the standard when managing normal or complicated delivery. (http://www.mchip.net/QoCsurveys). Merely measuring increased use of skilled attendants is not sufficient to guarantee that women receive appropriate management during labour and delivery. These findings further accentuate the need to pay due diligence to addressing quality as a key component of PBI schemes. In some studies, it is not possible to disentangle the impact of supply-side incentives from demand-side incentives, and little attention is paid to the issue of sustainability in any of the papers. The majority of studies are of short duration, making it hard to conclude whether the positive impact on the number of institutional deliveries will be sustained over time. It is also possible that other services that depend more on patients’ actions, such as decisions to present for ANC or family planning counselling, take longer time to show results and that studies of longer duration might show impact on these services (4).

However, while few studies of the impact of supply-side PBI on healthcare utilization or outcomes are structured as randomized controlled trials, we question whether this standard should be applied to the evaluation of a system changing initiatives, such as PBI. When PBI is implemented on the supply side, a number of systemic changes are introduced, along with the opportunity for providers and subnational levels of government managers, individually or in teams, to earn payments linked to performance in addition to their salary. These changes may include: clearly-defined expectations of strong performance, sometimes with targets for reaching some proportion of the population with priority services; clearly-defined reporting relationships from facilities to district teams; incentives at multiple levels that complement and strengthen the system; strengthened information systems; and stronger accountability at various levels. Implementing PBI is a package of system-wide interventions, and it is usually not possible to separate the impact, for example, of the additional money being received by the providers from the clarity and accountability for performance that comes from establishing metrics and holding people accountable for achieving these.

**Table 2.** Snapshot of delivery, ANC and quality metrics and impact

Country	Deliveries	ANC	Quality
Afghanistan (9)	Incentivized: Delivery care is provided according to Ministry of Health guidelines Impact: Increase in proportion of deliveries attended by skilled birth attendants−highest in WB's PBI areas, lower in comparison areas	Incentivized: Availability of ANC services Impact: Increase in proportion of pregnant women receiving first antenatal care visit−highest in WB's PBI areas, lower in comparison areas	Incentivized: Score on a balanced score care assessed input availability, equipment functionality, provider's knowledge, patients’ satisfaction Impact: Quality of care reportedly improved but meaning not clear in the paper
Bangladesh (5)	Incentivized: Two levels of target increases for numbers of deliveries established: 20% above baseline and 40% above baseline Impact: Average increases in upazilla health complexes were doubled and, in district hospitals, the rate grew 38% Note: In 2 districts, demand-side incentives were combined with supply-side incentives; in the third district, only supply-side incentives were introduced, and the fourth district was a control without supply or demand-side incentives	Incentivized: Two levels of target increases for ANC established: 20% above baseline and 40% above baseline Impact: Combined incentive model (S+D) had a larger effect on prenatal care	Incentivized: 100-point scale used in assessing quality. Both quality and quantity improvement targets needed to be reached for receivng performance-based payments Impact: Some reported changes include: introducing antenatal and postnatal care corners, increased readiness in labour room, breastfeeding corners, and post-operative rooms; installing a washroom adjacent to the labour room; and separating the sick newborn care unit within the paediatric ward of the district hospital
Cambodia (10)	Incentivized: Deliveries in a health facility Impact: Increase in institutional deliveries	Incentivized: ANC consultations Impact: The proportion of women who attended two or more antenatal sessions and had their blood pressure measured at least once remained the same at about 83%	Unclear
Democratic Republic of Congo (6)	Incentivized: Facility-based deliveries Impact: No change	Incentivized: ANC visits Impact: Not mentioned	Incentivized: Score on composite quality tool inflated payment (indicators not specified) Impact: Quality scores improved and patient-perceived quality improved
Egypt (8)	Incentivized: No Impact: None reported	Incentivized: Number of pregnant women receiving regular antenatal care visits compared to the total number of pregnant women in the catchment area Impact: Quality of ANC improved, i.e. ANC cases in PBI areas more likely to have a complete medical history taken, more complete examination undergone, and laboratory tests made than in the non-incentive scheme clinics (8)	Incentivized: Patients’ satisfaction rate, waiting time Impact: Nothing related to indicators above is listed but content of ANC improved
Haiti (11)	Incentivized: Assisted delivery by trained birth attendant Impact: Highly significant 17-27 percentage points increase in attended deliveries	Incentivized: Three ANC visits; HIV testing in ANC Impact: No change in ANC 3; No change in HIV testing in ANC	Incentivized: No Impact: Not available
Nepal (12)	Incentivized: Unit fees to facilities for each delivery attended at facility or home Impact: Increased chance of delivery in a government facility and reduced probability of home delivery No effect on C-section rate or on utilization of private delivery services	No effect on ANC visits (studied but not incentivized)	Incentivized: No Impact: Not available
Philippines (13)	Incentivized: The Women's Health Team receives US$ 21.73 for every delivery by a poor mother referred and assists during delivery at a health facility; mother given US$10.87 to cover childbirth-related expenses, such as transportation to facility and medicines, medical supplies, and food during her facility stay Impact: 7.6% increase in facility-based deliveries over the previous period	Incentivized: No Impact: Not available	Incentivized: No Impact: Not available
Rwanda (7)	Incentivized: Facility-based delivery Impact: Intervention facilities reported a significant 23% increase in the number of institutional deliveries	Incentivized: ANC 1; ANC 4; TT; IPT 2 Risky pregnancies referred during ANC Impact: No significant increase in ANC 4 measure; increase in the quality of ANC as measured by a composite quality index measure and provision of tetanus toxoid vaccine	Incentivized: Score on quality assessment deflates payment unless the score is 100% Impact: Not reported in paper

A practical approach to learning while doing is to design a progressive scale-up process (or ‘step-wise’ design), in which later initiators serve as controls for the early entrants. Scale-up could be phased in region by region because it is often politically unacceptable and operationally challenging to select intervention and control facilities in the same region. With a strategic approach to monitoring and evaluation during implementation and scale-up, a government could assess what's working and understand the aspects of design or implementation that need refinement. This approach would inform policy change when needed and enable governments to understand the impact of the performance-based incentive initiative.

Besides their varying levels of rigor, another weakness of the studies is that none provide enough detail on what was actually implemented to inform investments by donors or policy changes by governments or programmes. There is a growing recognition of the importance of documenting the PBI process–from generating buy-in among stakeholders to designing a programme, to implementation, learning from the programme, revision, and scale-up. Process documentation is a complement to reporting on quantitative evaluations since impact evaluations published in peer-reviewed journals typically do not provide insight into the ‘how’ and ‘why's of the observed changes because of the word-limits imposed by such journals (2). The studies reviewed here include only limited information on how incentivized performance measures are generated, including specific data sources and support to improve data-collection processes. These operational questions are of critical interest to governments, evaluators, practitioners, donors, and the global health community, both as a means to improve and revise programmes and to inform policy and are usually only available in unpublished final reports from the evaluator or donor.

It is clear from these studies and from the global evolution of PBI schemes that there is a growing recognition of the importance of stimulating improved quality as well as increasing the quantity of services provided. There are three categories of approaches that have been used in PBI programmes of developing countries to stimulate and reward improvements in quality that include the following (16):

- Making participation in a PBI scheme dependent on meeting service readiness criteria or linking incentive payments to the achievement or maintenance of accreditation levels
- Linking payment to adherence to clinical guidelines
- Assessing providers’ performance through the use of a quality checklist, index, or patients’ satisfaction survey, and either inflating performance payment or deflating it based on a score.

Some of the reviewed studies reward measures of the capacity to deliver quality services by assessing the quality of infrastructure, staff, medicines, and some processes. Some initiatives also attempt to incorporate clinical observations by supervisors into the quality tool that affects performance-based payments (5,7). For PBI to support quality measures effectively, there is a need for metrics of quality that can be routinely measured, reported and verified by a third party. In most settings, these potential measures are not included in country-specific health management information systems. For example, the use of a uterotonic in the third stage of labour and the use of magnesium sulphate to address pre-eclampsia/eclampsia are measureable indicators that are not often tracked. In addition to content of care indicators, there is an opportunity to learn from experiences in developed countries that are holding providers accountable by reducing or refusing reimbursement for avoidable hospital re-admissions.

### Limitations

A limitation of this paper and the presented evidence is that it draws narrowly from published literature that focuses on supply-side PBI for maternal and neonatal health and neglects the wider literature on supply-side PBI applied to other health services. In addition, this paper neglects the extensive literature on the effects of payment mechanisms on providers’ behaviour that is also directly relevant to understanding whether PBI can contribute to improving maternal and neonatal health outcomes. The paper on insurance in this series discusses the impact of paying providers a fee for each service on the caesarean section rate, especially when fees are considerably higher than for normal deliveries (17).

### Conclusions

Supply-side performance-based incentives—incentives to healthcare providers and managers at all levels—are being tried in many different ways in developing countries allover the globe. They are being incorporated into national public health delivery systems; social insurance schemes; contracts with service-delivery NGOs in fragile states; and the way providers are reimbursed in schemes that target safe deliveries. The majority of studies show that supply-side performance-based incentives are associated with increased numbers of institutional deliveries. However, their impact on other maternal and neonatal health services is inconclusive, although there is some evidence of impact on the content of ANC care. Moreover, while the numbers of institutional deliveries may have increased in PBI programmes, the impact on health outcomes is not clear, particularly on maternal and infant morbidity and mortality.

Because incentives exist in all health systems, considering how to align the incentives of the many health workers and their supervisors so that they focus efforts on achieving maternal and neonatal health goals makes intuitive sense. There are a wide range of models being developed and tested and still much remains to be learnt about what works best. More research is needed to help governments, donors, implementers, and others assess the cost-benefit of PBI's impact on maternal and child health relative to other approaches aimed at improving maternal and child health; more robust and rigorous quantitative studies are needed before firm conclusions about PBI's effectiveness can be drawn. Finally, more qualitative work on programme implementation is needed to help practitioners refine and improve their programmes.

In addition to understanding the impact of PBIs on health outcomes, there is a need to consider the impact of PBI on the functioning of health systems. For example, does the implementation of a programme that rewards for verified measures of performance enhance the quality and the use of information for decision-making, resulting in a stronger health information system? Does implementation of PBIs increase the productivity and effectiveness of human resources for health? Does the focus on rewarding results enable citizens to hold their health systems accountable? Does the opportunity to earn performance-based payments for enhanced use of health services provide the motivation and the funds needed to address shortages of essential medicines? Does the enhanced accountability for results strengthen supervision and arrangements in governance in the health systems?

Understanding how PBI initiatives introduce systemic changes into health systems is a potentially valuable addition to the agenda for future research.
